# NMR assignments of the human homodimeric mitochondrial ATP synthase inhibitor IF1

**DOI:** 10.21203/rs.3.rs-7944890/v1

**Published:** 2025-11-06

**Authors:** Julia Jerolamon-Martínez, Nathan Alder, Andrei Alexandrescu

**Affiliations:** University of Connecticut; University of Connecticut; University of Connecticut

**Keywords:** ATP synthase, ATPase inhibitor, coiled-coil dimer, pH-dependent oligomer, mitochondria

## Abstract

ATPase inhibitory factor 1 (IF1) is the only known endogenous, proteinaceous inhibitor of mitochondrial ATP synthase in mammals. The inhibitor forms an antiparallel coiled-coil, which binds ATP synthase through an N-terminal a-helix extension that is disordered in the free protein. Because the IF1 dimer affects mitochondrial bioenergetics through its modulation of ATP synthase, it is a therapeutic target for cancer and cardiac disease. Here, we report ^1^H, ^13^C and ^15^N NMR assignments for the mature dimeric form of human IF1. Secondary structure analyses based on chemical shifts and short-range NOE patterns indicate the N-terminal half of the 81-residue IF1 is intrinsically disordered, while the C-terminal half adopts a continuous α-helix. The chemical shift assignments for human IF1 provide a foundation for future mechanistic structure-function studies and NMR-based drug screening.

## Biological context

The ATPase Inhibitory Factor 1 (henceforth IF1) is a key mediator of mitochondrial bioenergetics during metabolic stress (Garcia-Bermudez and Cuezva 2016). IF1, which forms an antiparallel coiled-coil homodimer, is an endogenous inhibitor of ATP synthase - the transmembrane mitochondrial proton gradient-driven enzyme that synthesizes ATP during steady-state oxidative phosphorylation. Severe metabolic stress conditions such as hypoxia induce mitochondrial depolarization, leading ATP synthase to operate in reverse as a proton-motive motor, hydrolyzing ATP to re-establish the proton gradient. Under these conditions, IF1 can bind to the ATP synthase active site and act as a “brake” for ATP hydrolysis. This limits excessive ATP consumption and maintains cellular energy levels (Bason et al. 2011). In addition to its inhibitory role, IF1 preserves the cristae architecture of the inner mitochondrial membrane by stabilizing ATP synthase oligomers upon binding. This is important for mitochondria-mediated apoptosis, since the process requires the release of cytochrome c into the cytosol, which entails remodeling of the inner membrane. The IF1-ATP synthase complex helps prevent such remodeling, reducing apoptosis (Faccenda et al. 2013). Taken together, the functional and structural roles of IF1 make it a prosurvival factor, beneficial for acute stress situations such as ischemia-reperfusion injury (Wu et al. 2022).

IF1 plays context-dependent roles in heart physiology. IF1 is cardioprotective against acute ischemia/early reperfusion. Sustained IF1 activity with prolonged cardiac stress can be deleterious, however, as shown by IF1 knock-out heart models protected from pressure overload-induced cardiac hypertrophy (Wu et al. 2022; Zhou et al. 2022). Furthermore, IF1 overexpression/knockout studies in mouse neurons showed IF1 enhances synaptic transmission and cognition (Esparza-Molto et al. 2021).

IF1 may also enable tumor-cell survival under chronic hypoxic stress in certain cancers (Dominguez-Zorita and Cuezva 2023; Wu et al. 2022). IF1 expression is upregulated across many cancers, yet its prognostic significance is tumor-type-specific. For example, IF1 overexpression in glioma, liver, and bladder cancer is linked to increased tumor progression. By contrast, high IF1 levels in lung, colon, and breast cancer are linked to non-metastatic phenotypes (Dominguez-Zorita and Cuezva 2023).

The human IF1 gene *ATP5IF1* (UniProt Q9UII2) consists of a mitochondrial targeting sequence (residues 1–25) and the IF1 inhibitor (residues 26–106). Post-translational cleavage of the transit peptide yields the 81-residue mature IF1 fragment (9.5 kDa monomer molecular weight), the subject of the present study. The numbering scheme for the mature inhibitor IF1 (1–81) is used throughout this paper. The IF1 fragment forms an antiparallel coiled-coil, with dimeric and tetrameric oligomerization states modulated by solution pH. Acidic matrix pH when the inner membrane is uncoupled or the electron transport chain is compromised, favors an antiparallel coiled-coil dimer formed through the C-terminal half of the protein (residues 40–81) that leaves the unfolded N-terminal half (residues 1–40) poised to bind and inhibit ATP synthase (Boreikaite et al. 2019; Gordon-Smith et al. 2001). By contrast, basic pH favors tetramers that are prevalent in energized mitochondria and are assembled through the N-terminus, making this region unavailable for ATP synthase inhibition (Cabezon et al. 2001). Ligands that shift the IF1 dimer–tetramer equilibrium provide a route to pharmacological control of ATP synthase, positioning IF1 as a compelling therapeutic target.

Previous NMR work reported NMR chemical shift assignments and a solution structure of the C-terminal 44–84 segment of bovine IF1 lacking the N-terminal inhibitory region. The bovine and human C-terminal coiled-coil segments share 79% sequence identity. A second NMR study investigated the monomeric unfolded 1–40 segment of human IF1 lacking the coiled-coil domain but provided only a small number of chemical shift assignments for this segment (Galber et al. 2023). Here, we report ^1^H, ^15^N, and ^13^C NMR chemical shift assignments as well as secondary structure information for the full-length 1–81 mature human IF1. NMR work on the protein presented challenges due to a mixture of IDP (1–39) and regular a-helical structure (40–81), limited chemical shift dispersion, and a high fraction of similar long-chain aliphatic residues in the sequence (14 Glu, 10 Lys, 8 Arg). We anticipate our assignments will be useful for mechanistic and drug-binding studies of IF1.

## Methods and experiments

### Cloning, expression, and purification of IF1

The human *ATP5IF1* gene encoding residues G26-D106 (1–81 in the numbering scheme of mature IF1, with codons optimized for expression in *Escherichia coli*), preceded by a 6x-histidine tag and a TEV protease cleavage site, was cloned into a pET-28a vector (Blue Heron Biotechnology, Bothell, WA). The construct was verified by whole-plasmid sequencing using Oxford Nanopore Technology (Plasmidsaurus, Louisville, KY). The plasmid was transformed into *E. coli* C41 (DE3) cells, and 500 mL cultures were grown at 37°C in M9 minimal media (47.7 mM Na_2_HPO_4_, 22 mM KH_2_PO_4_, 8.6 mM NaCl, 2 mM MgSO_4_, 5 μM FeCl_3_, 5 μM CaCl_2_) supplemented with 4 g/L 1-^13^C-D-glucose (4 ml/L glycerol for non-^13^C-labeled samples) and/or 1 g/L ^15^NH_4_Cl. Kanamycin (25mg/L) was included in the growth medium to maintain selection of the pET expression vector. Expression was induced at OD_600_ = 0.6 with 0.64 mM isopropyl thio-β-D-galactoside (IPTG), and cells were grown for an additional 20 hours at 25°C. The cells were harvested by sedimentation at 3,470 g using a Sorvall Lynx 6000 fixed-angle rotor (Thermo Fisher Scientific, Waltham, MA), and the pellets were flash frozen and stored at −80°C.

All subsequent purification steps were performed at 4°C. Frozen pellets from a 500 mL culture were resuspended in 20 mL of lysis buffer: 25 mM potassium phosphate buffer pH 6.4, 20 mM Imidazole, and 1x cOmplete^™^ EDTA-free protease inhibitor cocktail (Roche, Basel Switzerland). The pH after imidazole addition was 7.4. Cells were lysed by sonication on a Q700 Sonicator (Qsonica, Newtown, CT) with instrument parameters of 40 s on/20 s off cycles at 70% amplitude, and 3 min on-time total. After sonication, the cell extract was clarified by sedimentation (30,000 g, 45 min). NaCl was added to the supernatant to a concentration of 0.2 M, and the mixture was incubated with 2-ml pre-equilibrated Ni-NTA agarose (QIAGEN GmbH, Hilden, Germany).

All subsequent purification buffers were adjusted to pH 6.4, which significantly reduced IF1 aggregation. The Ni-NTA resin was washed with 7 column volumes of 30 mM imidazole followed by 7 column volumes of 40 mM imidazole in buffer A (25 mM potassium phosphate buffer, pH 6.4, 0.2 M NaCl). IF1 was eluted with 5 column volumes of 500 mM imidazole in buffer A. The eluate was buffer-exchanged using an Amicon^®^ Ultra centrifugal filter, 3 kDa MWCO (MilliporeSigma, Burlington, MA) into buffer A. 1 mM DTT and recombinant His_6_-TEVsh protease (1:15 (w/w) TEV:IF1) were added to the retentate. The TEVsh protease was prepared in-house as described in the literature (van den Berg, Lofdahl et al. 2006). The TEVsh plasmid, Addgene plasmid # 125194 (Watertown, MA), was a gift from Helena Berglund. TEVsh protease cleavage was done overnight at 4°C with gentle shaking. After sedimentation to remove aggregates (10 min at 10,000 g), the supernatant was incubated with Ni-NTA to capture his_6_-TEV protease. The flowthrough containing tag-free IF1 was buffer exchanged using Amicon^®^ centrifugal filtration (3 kDa MWCO) into buffer A. Protein concentrations were determined using the Qubit^™^ Protein Assay Kit on a Qubit^™^ 4 Fluorometer (Thermo Fisher Scientific, Waltham, MA) following the manufacturer’s instructions. Purified IF1 samples were stored at 4°C and were used for NMR within 24h of purification.

### NMR spectroscopy

NMR experiments were performed at 37°C on a Bruker AVANCE NEO 600 MHz spectrometer equipped with a cryogenic probe. Samples contained 0.1–0.4 mM IF1 monomer in 25 mM potassium phosphate buffer and 200 mM sodium chloride. In the first phases of the project, we optimized the sample pH and temperature for NMR experiments. The best NMR spectra were obtained over a narrow range of sample conditions, between about pH 4.7 and 5.6, and temperatures between 37 and 41 degrees. More basic pH conditions led to loss of peaks due to oligomerization into tetramers and higher-order aggregates, while more acidic conditions unfolded the protein. Similarly, temperatures below 37 °C shifted the equilibrium to oligomers, whereas higher temperatures caused losses of amide protons due to hydrogen exchange and eventually protein unfolding. Thus, all NMR experiments were performed at pH 5.3–5.6 and 37°C.

**Figure 1:**
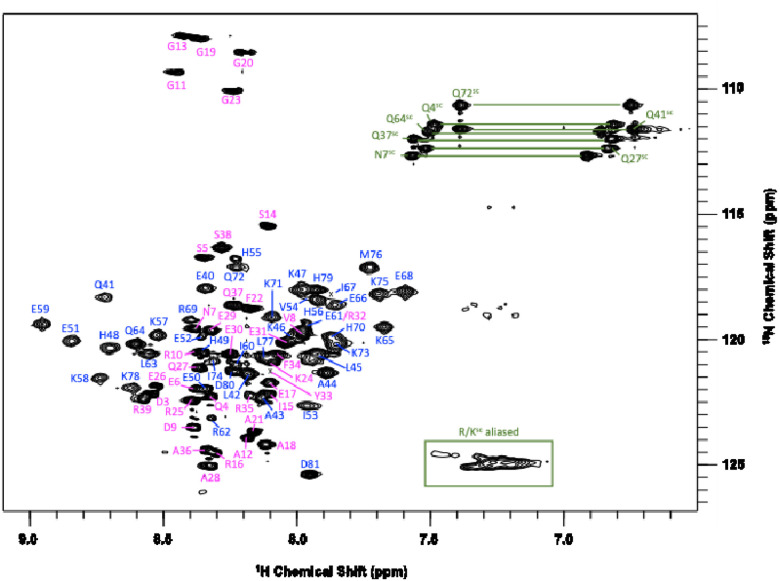
^1^H-^15^N SOFAST-HMQC spectrum of dimeric human IF1 at 600 MHz, 37°C. The protein monomer concentration was 445 μM in 25 mM potassium phosphate and 200 mM NaCl, pH 5.3. Peaks from the disordered N-terminus (residues 1–38) are colored pink; peaks from the helical coiled-coil region (39–81) are blue. Asn/Gln side-chain amide resonances are indicated with horizontal green lines, and an aliased group of sidechain crosspeaks due to Arg/Lys residues is enclosed in a green box.

Backbone resonance assignments for the IF1 dimer were determined from u-[^15^N,^13^C]-IF1 samples (110 μM, pH 5.3, 10% D_2_O) using 3D HNCACB, HNCA, HN(CO)CA, HNCO, and HN(CA)CO spectra. The sample was lyophilized and resuspended in D_2_O (pH 5.6) for ^1^H-^13^C experiments: 2D ^1^H-^13^C HSQC and 3D HCCH-TOCSY, CCH-TOCSY, ^1^H-^13^C NOESY (120 ms mixing time). A separate u-[^15^N]-IF1 sample (445 μM, pH 5.3, 5% D_2_O) was used for acquiring ^1^H-^15^N TOCSY HSQC (70 ms mixing time) and ^1^H-^15^N NOESY-HSQC (120 ms mixing time) spectra. This sample was also used for the “fingerprint” ^1^H-^15^N SOFAST-HMQC in Fig. 1. Protein integrity between NMR spectra was checked with serial ^1^H-^15^N SOFAST-HMQC spectra. ^1^H shifts were referenced directly to internal DSS (2,2-dimethyl-2-silapentane-5-sulfonate), whereas ^13^C and ^15^N were referenced indirectly (Wishart et al. 1995).

### Assignments and data deposition

An annotated ^1^H-^15^N SOFAST-HMQC spectrum for dimeric IF1 is shown in Fig. 1. Backbone amide (H^N^-N) assignments are 98% complete, and assignment coverage is also 98% complete for all backbone atoms. The only residues missing H^N^-N assignments are Gly1 and Ser2, which experience fast amide proton exchange with solvent. Assignment coverage statistics for all NMR-active atoms are ^1^H (86%), ^13^C (77%), ^15^N (64%). The somewhat low percentages for ^13^C and ^15^N are due to the presence of a large fraction of Lys, Arg, and Glu residues in the protein sequence.

**Figure 2:**
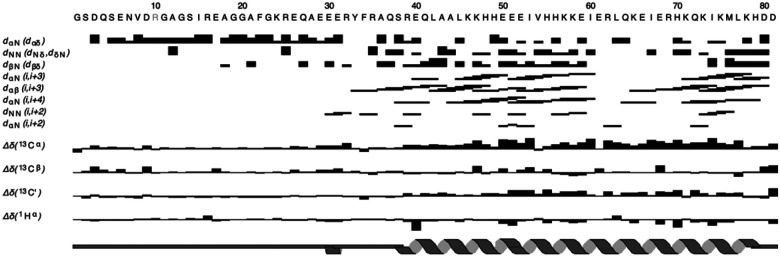
Secondary structure analysis of dimeric human IF1 derived from backbone chemical shifts with the program **DANGLE** (CcpNmr Analysis v2.5.2) (Cheung et al. 2010; Vranken et al. 2005) and corroborated by diagnostic NOE patterns in the ^1^H-^15^N and ^1^H-^13^C NOESY-HSQC spectra. The consensus secondary structure obtained from DANGLE is at the bottom of the figure. The N-terminal segment (residues 1–38) is disordered, followed by a continuous alpha-helical region (residues 39–80).

Our assigned chemical shifts and preliminary analysis of ^1^H-^15^N NOESY-HSQC and ^1^H-^13^C NOESY-HSQC spectra, were used to calculate a consensus secondary structure for IF1 (Fig. 2) using the DANGLE subroutine (Cheung et al. 2010) of the CcpNmr Analysis v2.5.2 suite of programs (Cheung et al. 2010). The IF1 dimer consists of an a-helical region between about residues R39-D81, although a few weak NOEs characteristic of a-helical structure extend to about E30. Upstream of E30 towards the N-terminus, the NMR data do not support a-helical structure giving intense NMR signals characteristic of long T2 values and NOEs to solvent, both typical of unstructured conformations. The secondary structure pattern observed for human IF1 in the present study is consistent with the previously described antiparallel coiled-coil structure of bovine IF1 (residues 44–84), and the observation that residues 10–48 corresponding to the inhibitory segment are unstructured in solution (Gordon-Smith et al. 2001).

## Data Availability

The chemical shift data from this study have been deposited in the Biological Magnetic Resonance Data Bank (BMRB) under the accession number 53396.
